# Bottled Wrath

**Published:** 1859-06

**Authors:** 


### BOTTLED WRATH

Like pent up steam is all the better and safer for being "let off." If a little boy "stumps his toe," he grits his teeth, hisses out a malediction, gives the offending stone a savage kick, and straitway feels better. A groan is the healthful vent, the anodyne of pain; and tears relieve and save the almost breaking heart. It is he who cannot cry, who dies with sorrow. There is neither sense nor safety in uncomplaining suffering, as to the repression of its instinctive exhibition. There is no pain where there is no nerve. The more nerve, the greater the susceptibility to suffering. The nervous influence, as it is called, is a fluid, just as blood is a fluid; and as the blood flowing along the blood vessels gives life, so the nervous influence flowing along the nerve channels gives sensation. If the blood has no outlet, we become diseased in a few hours. If a wound is inflicted, it will get well ordinarily the sooner, if blood flows or is taken. So in the infliction of pain, it is relieved instantly, if the nervous influence has vent. That vent, that scapement, that water-way, is in every movement of a muscle, in every wink of the eye, in every crook of the finger, in every thought we think; for we can no more think, or move, or feel, without the expenditure of nervous influence, than would a telegraphic record be made without the expenditure of electricity, or the locomotive would move an inch without the consumption of an amount of steam.

If then pain is inflicted as to mind or body, the sooner we can give an outlet to the nervous influence, the more immediate will be the relief; therefore nature in her philosophy, has implanted an instinct which complains on the very instant that harm is done; hence the groan, the cry, the shriek, and these before second thoughts have time to come and whisper it is not dignified to cry, or shriek or groan ; and many an one has exclaimed in mortification, at the supposed weakness of so doing, "what a fool to have made such a racket!" So it does seem that at almost every turn of life, we attempt to thwart wise nature, and hedge her up by bald reason, in her attempt to soothe and save. To put all this in plainer phrase, the louder you groan in sickness and suffering, the sooner will you get well. Hence to a certain extent, when a person complains a great deal, we have fallen into the habit of saying, oh! there's not much the matter with him, he is more scared than hurt. We have insensibly fallen into the habit of drawing such conclusions, because we have noticed that persons who complain a great deal, complain a long time, they don't die, and very often get well in a few days.

And here let us make an earnest appeal for infancy and early childhood. When a child is hurt, never hush it up ; it is an inexcusable barbarity ; it is fighting against nature ; it is repressing her instincts ; and for the same reason, if physical punishment is inflicted on a child, never repress its crying ; it is a perfect brutality ; cases are on record where children have been thrown into convulsions in their efforts to silence ; and very little less hurtful is it to hire them to silence. A thousand fold better is it to soothe by kindly words, and acts ; and divert the mind by telling stories, or by explaining pictures, or by providing with new toys. We have many a time in our professional experience as to sick children, found more benefit to be derived from a beautiful or interesting toy, than from a dose of physic. The greatest humanity a mother can exhibit in respect to her sick child is to *divert it*, DIVERT IT, DIVERT IT, in all the pleasing ways possible, as we ourselves, who are larger children, feel some times really sick, when a cheerful faced and much loved friend has come in, and before we knew it, we had forgotten that anything was the matter with us.

We have sadly wandered from what we intended, when the heading of this article was written, and not to detain the reader longer, we will sum up as concisely as possible, that if any man has a fretful wife, one who does not fail to greet him on his return from the business of the day, by pouring out her complaints with overwhelming volubility, who never sits down to a family meal without some whine or doggish growl, let him adopt the following plan for letting out the "bottled wrath," before he comes in gun shot of home.

Let one of the servants be very little, very lazy, very fat, and very stupid, in fact pretty much of a fool. Such a girl can no more be excited into a passion than she could be stimulated to hurt herself by hard work, and she will bear a great deal of verbal pummelling. You can't make her saucy. She is too lazy to give "warning," and too wise to get mad, for there is no fool but has some redeeming quality; she will stand the fire of verbal abuse by the hour, for she knows words don't hurt. So while the boy kicks the stone, let the wife blaze away at the lump of dough, and all the amunition being expended before you get home, the steam being exhausted, "re-action" takes place, and the hyena of high noon will be a lamb at sun down, at the tea table, at the parlor fire, and at bed time.

